# Microbiota alterations in patients treated for susceptible or drug-resistant TB

**DOI:** 10.5588/ijtldopen.24.0325

**Published:** 2024-08-01

**Authors:** M. Hauptmann, B. Kalsdorf, J.E. Akoh-Arrey, C. Lange, U.E. Schaible

**Affiliations:** ^1^ Priority Area Infections, Division of Cellular Microbiology,; ^2^ Outpatient Pulmonology Ward, and; ^3^ Division of Clinical Infectious Diseases, Research Center Borstel, Leibniz Lung Center, Borstel, Germany.; ^4^ Respiratory Medicine and International Health, University of Lübeck, Lübeck, Germany;; ^5^ Baylor College of Medicine and Texas Children´s Hospital, Global TB Program, Houston, TX, USA;; ^6^ German Center for Infection Research (DZIF), Partner Site Hamburg-Lübeck-Borstel-Riems, Borstel, Germany;; ^7^ Biochemical Microbiology & Immunochemistry, University of Lübeck, Lübeck, Germany

**Keywords:** *Mycobacterium*, nosocomial pneumonia

## Abstract

**BACKGROUND:**

We investigated alterations of human microbiota under anti-TB therapies in relationship to the level of *Mycobacterium tuberculosis* drug response.

**METHODS:**

Stool, sputum, and oral swab samples were analysed from participants with treatment-naïve TB and participants treated for drug-susceptible TB (DS-TB), drug-resistant TB without injectable drugs (DR-TB-inj–), or with injectable drugs (DR-TB-inj+) at 27–42 days of therapy.

**RESULTS:**

From September 2018 to December 2019, 5 participants with treatment-naïve TB, 6 participants with DS-TB, 10 participants with DR-TB-inj–, and 4 participants with DR-TB-inj+ were recruited. Reduced alpha diversities in stool samples indicated more profound dysbiosis in participants treated for DR-TB than in participants treated for DS-TB (–12% (non-significant) for DS-TB, –44% (*P* < 0.001) for DR-TB-inj–, and –60% (*P* < 0.05) for DR-TB-inj+ compared to treatment-naïve participants). While reduced abundances were observed in numerous taxa, genus *Lactobacillus* revealed the most substantial abundance increase in sputa of participants treated for DR-TB compared to treatment-naïve ones (*P* < 0.05 for DR-TB-inj– and DR-TB-inj+). Notably, a group of nosocomial pneumonia-associated taxa was increased in oral swabs of the DR-TB-inj+ compared to the treatment-naïve group (*P* < 0.05).

**CONCLUSIONS:**

Second-line anti-TB therapy in participants with DR-TB results in altered microbiota, including reduced alpha diversity and expansion of phylogenetically diverse taxa, including pathobionts.

## RÉSUMÉ

**CONTEXTE:**

Nous avons étudié les altérations du microbiote humain sous traitements anti-TB en lien avec le niveau de réponse médicamenteuse de *Mycobacterium tuberculosis*.

**METHODES:**

Des échantillons de selles, d'expectorations et d'écouvillons buccaux ont été examinés chez des participants n'ayant jamais reçu de traitement contre la TB et des participants traités pour une TB sensible aux médicaments (DS-TB), une TB résistante aux médicaments sans médicaments injectables (DR-TB-inj–), ou avec des médicaments injectables (DR-TB-inj+) après 27 à 42 jours de traitement.

**RESULTATS:**

De septembre 2018 à décembre 2019, 5 participants atteints de TB naïve, 6 participants atteints de DS-TB, 10 participants atteints de DR-TB-inj– et 4 participants atteints de DR-TB-inj+ ont été sélectionnés. La diminution des diversités alpha dans les échantillons de selles indique une dysbiose plus profonde chez les participants traités pour la DR-TB que chez les participants traités pour la DS-TB (–12% (non significatif) pour la DS-TB, –44% (*P* < 0,001) pour la DR-TB-inj–, et -60% (*P* < 0,05) pour la DR-TB-inj+ par rapport aux participants n'ayant jamais reçu de traitement). Bien que des abondances réduites aient été observées dans de nombreux taxons, le genre Lactobacillus a montré la plus forte augmentation d'abondance dans les expectorations des participants traités pour la DR-TB par rapport aux participants non traités (*P* < 0,05 pour DR-TB-inj– et DR-TB-inj+). En particulier, un groupe de taxons associés à la pneumonie nosocomiale était plus abondant dans les écouvillons oraux du groupe DR-TB-inj+ par rapport au groupe non traité (*P* < 0,05).

**CONCLUSIONS:**

Le traitement anti-TB de deuxième ligne chez les participants atteints de DR-TB entraîne des changements dans le microbiote, notamment une diminution de la diversité alpha et une expansion de taxons phylogénétiquement diversifiés, y compris les pathobiontes.

TB is the leading cause of death from a bacterial infectious disease worldwide.^1^ Reaching the goals of the WHO End TB Strategy, aiming for a 95% reduction in TB incidence and a 90% reduction in TB-attributed mortality, and zero families suffering catastrophic costs related to TB by 2035,^2^ is challenged by the emergence of drug-resistant strains of the *Mycobacterium tuberculosis* complex.^3^

Multidrug-resistant TB (MDR-TB) is defined by the resistance of *Mycobacterium tuberculosis* to both rifampicin and isoniazid. However, resistance to rifampicin alone (RR) is considered a surrogate for MDR-TB, collectively referred to as MDR/RR-TB.^4,5^ Until 2021, extensively drug-resistant TB (XDR-TB) was defined as MDR/RR-TB plus resistance against a later-generation fluoroquinolone (i.e. levofloxacin and/or moxifloxacin) and resistance against a second-line injectable agent (amikacin, capreomycin, and/or kanamycin).^6^ In 2021, these definitions were revised, with pre-XDR-TB being defined as MDR/RR-TB plus resistance to a later generation fluoroquinolone and XDR-TB being defined as pre-XDR-TB plus resistance of *M. tuberculosis* against bedaquiline and/or linezolid.^7,8^

The gut microbiota is one of the densest ecosystems of microorganisms known, with an estimated 4 x 10^13^ bacteria.^9^ Firmicutes, Bacteroidetes, and Actinobacteria are the dominant bacterial phyla.^9,10^ The respiratory tract, especially the deeper lung tissue, harbours a lower number of bacteria, forming consortia, which are highly dynamic and at least partly influenced by a constant exchange with the oral cavity microbiota.^11^

The treatment of drug-resistant TB is unique because of the number and types of antibiotics and the long duration of therapy, which affect the diversity and composition of the human intestinal microbiota, as shown in several recent publications.^12,13^ Second-line anti-TB treatment includes several broad-spectrum antibiotics, especially the injectable aminoglycosides and carbapenems. To our knowledge, no study has investigated the effect of different second-line anti-TB treatment regimens for persons with varying levels of drug-resistant TB on the diversity of human microbiota.

To evaluate the changes in the microbiota during broad-spectrum antibiotic treatment, we compared the composition of microbiota from sputum, oral swabs, and stool of persons with TB, which were either treatment-naïve or treated with first-line, second-line, or second-line injectable drug regimens at the initiation of therapy and following 4–6 weeks of treatment.

## MATERIAL AND METHODS

Detailed methods for sample collection, 16S rRNA sequencing, and statistics are provided in the supplementary material.

### Participants

Participation in this study was offered to all patients over 18 years of age, without the need for legal guardianship, who presented with culture-confirmed TB at the Medical Clinic of the Research Centre Borstel – Leibniz Lung Centre between September 2018 and December 2019. All patients who provided written consent were recruited. The treatment regimens were selected based on the results of genotypic prediction of drug resistance by GeneXpert (Cepheid, Sunnyvale, CA, USA), GenoType^®^ MTBDR*plus* (Hain Lifescience, Nehren, Germany) and GenoType^®^ MTBDRsl (Hain Lifescience), and phenotypic drug resistance testing on liquid media using MGIT (Becton Dickinson, Franklin Lakes, NJ, USA). Cases were classified as being either treatment-naïve or as receiving treatment for DS-TB, MDR/RR-TB using only oral drugs (DR-TB-inj–), or MDR/RR-TB using a combination of oral and injectable drugs (DR-TB-inj+).

### Ethics

The study protocol was reviewed and approved by the University of Lübeck Ethics Committee, Lübeck, Germany (AZ 18–280). All participants provided written informed consent.

## RESULTS

### Treatment regimens and study groups

**Figure 1.**
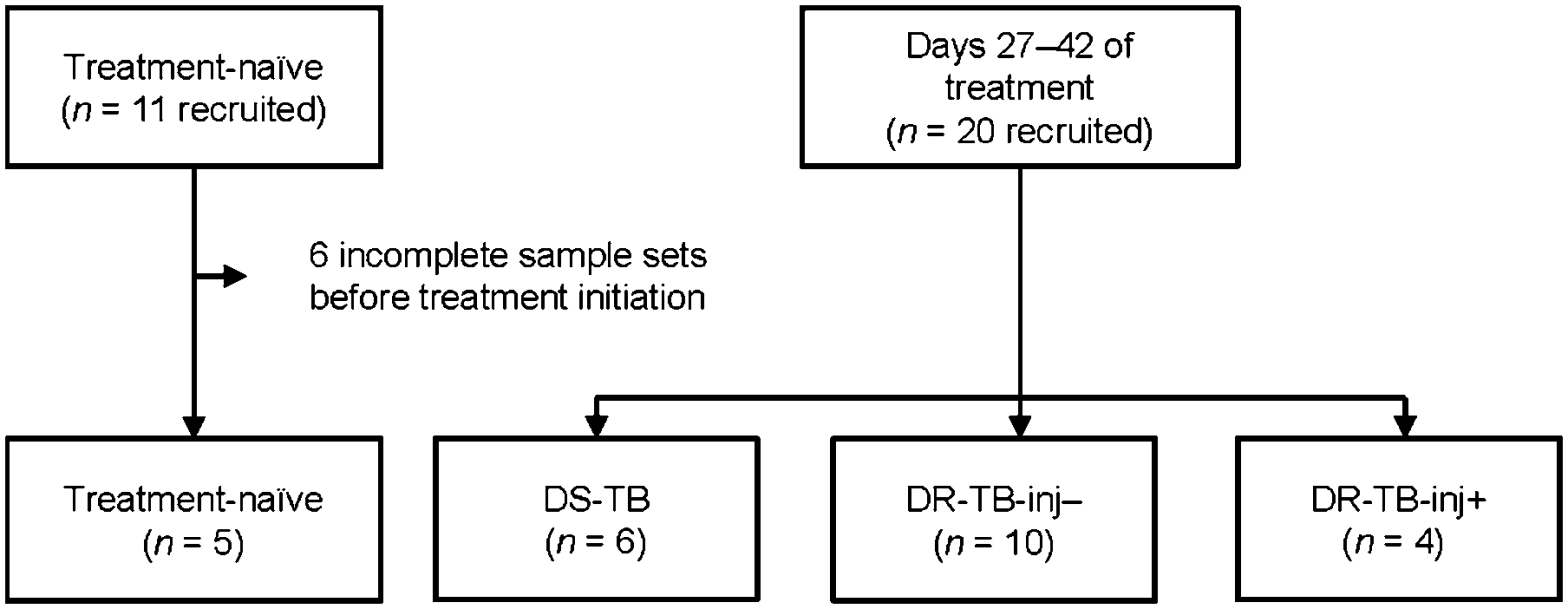
Flow chart of patient recruitment. DS-TB = drug-susceptible TB; DR-TB-inj– = drug-resistant TB treated without injectable agents; DR-TB-inj+ = drug-resistant TB treated with injectable agents (meropenem and/or amikacin).

Eleven persons with treatment-naïve TB were recruited. Of those, five provided all sample types before the start of the treatment; the other six were excluded. Twenty persons with TB were recruited 27–42 days post-treatment initiation. Of these, one patient (P17) did not provide a stool sample at 27–42 post-treatment start, and the data from the oral swab of P20 were excluded due to low 16S sequence coverage. Of the 20 persons with TB, 6 received treatment for DS-TB (including 1 patient who received levofloxacin instead of pyrazinamide due to treatment-naïve hepatopathy), 10 received treatment for DR-TB-inj–, and 4 received DR-TB-inj+ treatment (of those 4, all received meropenem and one additionally received amikacin and delamanid). The 5 treatment-naïve persons with TB were additionally recruited at 27–42 days post-treatment start (2 in the DS-TB group, 1 in the DR-TB-inj–, and 2 in the DR-TB-inj+ group (Figure 1 and Supplementary Figure S1). There were no apparent differences in median age, body mass index (BMI), sex distribution, or prevalence of comorbidities between the study groups (Table). Participants were acutely ill with a Karnofsky index of 50–90% due to the newly diagnosed infection with *M. tuberculosis*.

**Table.** Study demographics and comorbidities.

		Day 27–42 of treatment		
	Treatment-naive (*n* = 5)	DS-TB (*n* = 6)	DR-TB-inj–(*n* = 10)	DR-TB-inj+(*n* = 4)
Age, years, median [IQR]	33 [31–35]	42 [34.2–49]	27 [20.2–40.8]	31.5 [29.8–32.8]
BMI, kg/m^2^, median [IQR]	22.8 [21.8–27.4]	20.4 [19–26.7]	20.4 [18.9–23.4]	22.3 [21.3–23.9]
Female sex, %	20.0	16.7	10.0	0.0
Comorbidities, %				
HIV	0	0	0	0
Type 2 diabetes	0	0	0	0
Hepatitis B (current)	0	0	0	0
Hepatitis C (chronic)	0	0	10	0
Hepatitis C status unknown	0	0	10	0
Lymphopenia of unknown origin	0	16.7	0	0
Other immunosuppression	0	0	0	0

DS-TB = drug-susceptible TB; DR-TB-inj– = drug-resistant TB without injectable drugs; DR-TB-inj+ = drug-resistant TB with injectable drugs; IQR = interquartile range; BMI = body mass index.

### Microbiota diversity

**Figure 2.**
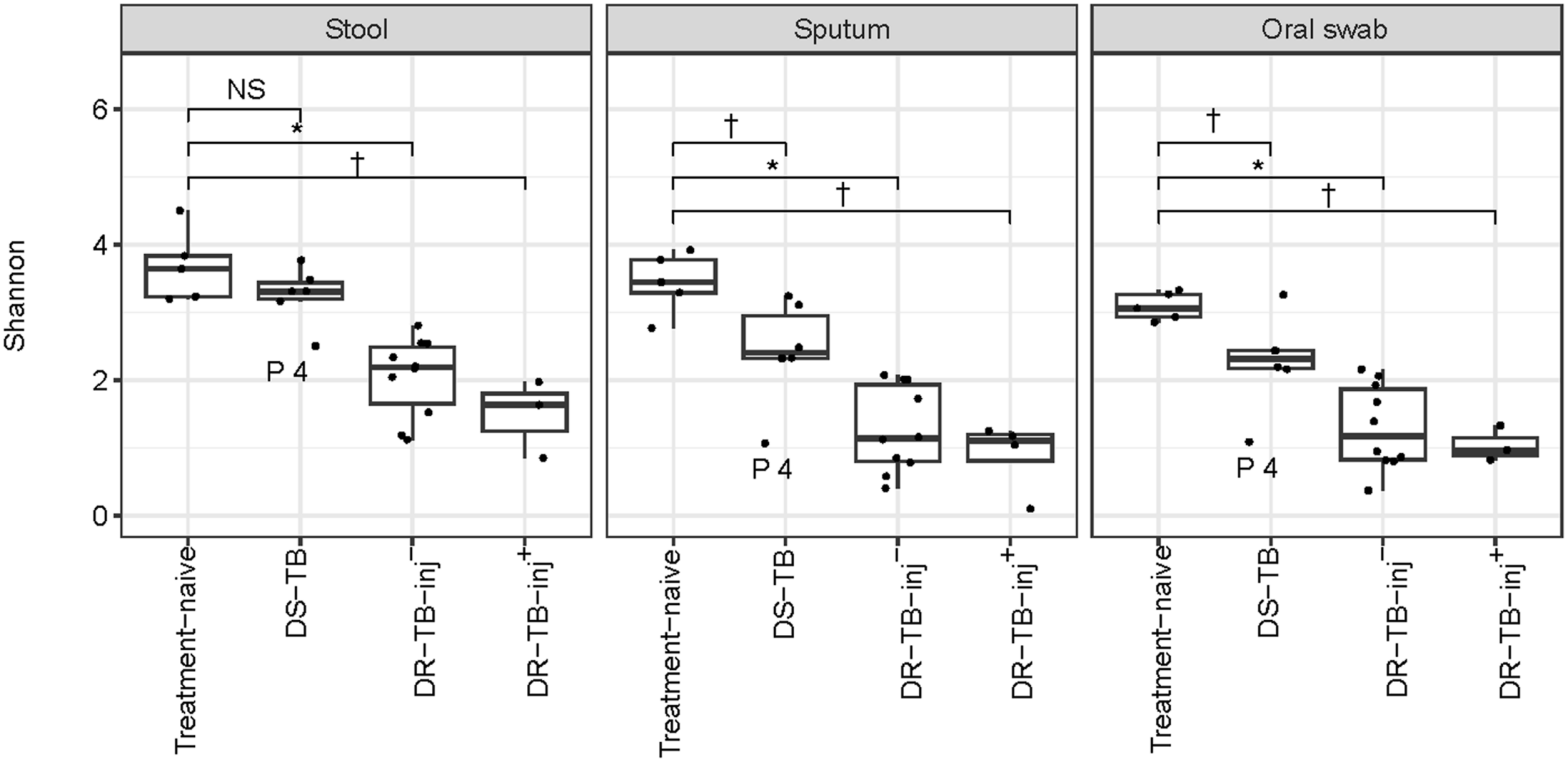
Alpha diversity. Treatment of participants with DS-TB caused a small decrease in Shannon diversity in stool, sputum, and oral swab specimens, while both DR-TB-inj– and DR-TB-inj+ treatments had a stronger impact on Shannon diversity. Pairwise comparisons were performed using Wilcoxon signed-rank tests. Data from 5 participants with treatment-naïve TB, 6 participants with DS-TB, 10 participants with DR-TB-inj-, and 3–4 participants with DR-TB-inj+ are shown. * *P* < 0.001. ^†^ *P* < 0.05. NS = non-significant; DS-TB = drug-susceptible TB; DR-TB-inj– = drug-resistant TB treated without injectable agents; DR-TB-inj+ = drug-resistant TB treated with injectable agents (meropenem and/or amikacin).

Compared to treatment-naïve controls, anti-TB treatment resulted in a 12% (non-significant), 44% (*P* < 0.001), and 60% (*P* < 0.05) decrease in the alpha diversities in stool samples of participants in the DS-TB, DR-TB-inj–, and DR-TB-inj+ groups, respectively. In sputum and oral swab specimens, the percentage decreases in alpha diversity compared to treatment-naïve controls were highest in participants of the DR-TB-inj+ group, followed by the DR-TB-inj– and DS-TB groups (sputum: 74% (*P* < 0.05), 63% (*P* < 0.001), 30% (*P* < 0.05); oral swab: 66% (*P* < 0.05), 58% (*P* < 0.001), 27% (*P* < 0.05), respectively) (Figure 2).

Community compositions in specimens from oral swabs, sputum, and stool samples were similar between persons with treatment-naïve TB and those treated for DS-TB, while compositions were different under DR-TB treatment with both all-oral and injectable drug regimens (permutational analysis of variance resulted in *P* < 0.001 for all collection sites, comparing community compositions by ‘treatment group’) (Supplementary Figure S2).

### Differentially abundant taxa

**Figure 3.**
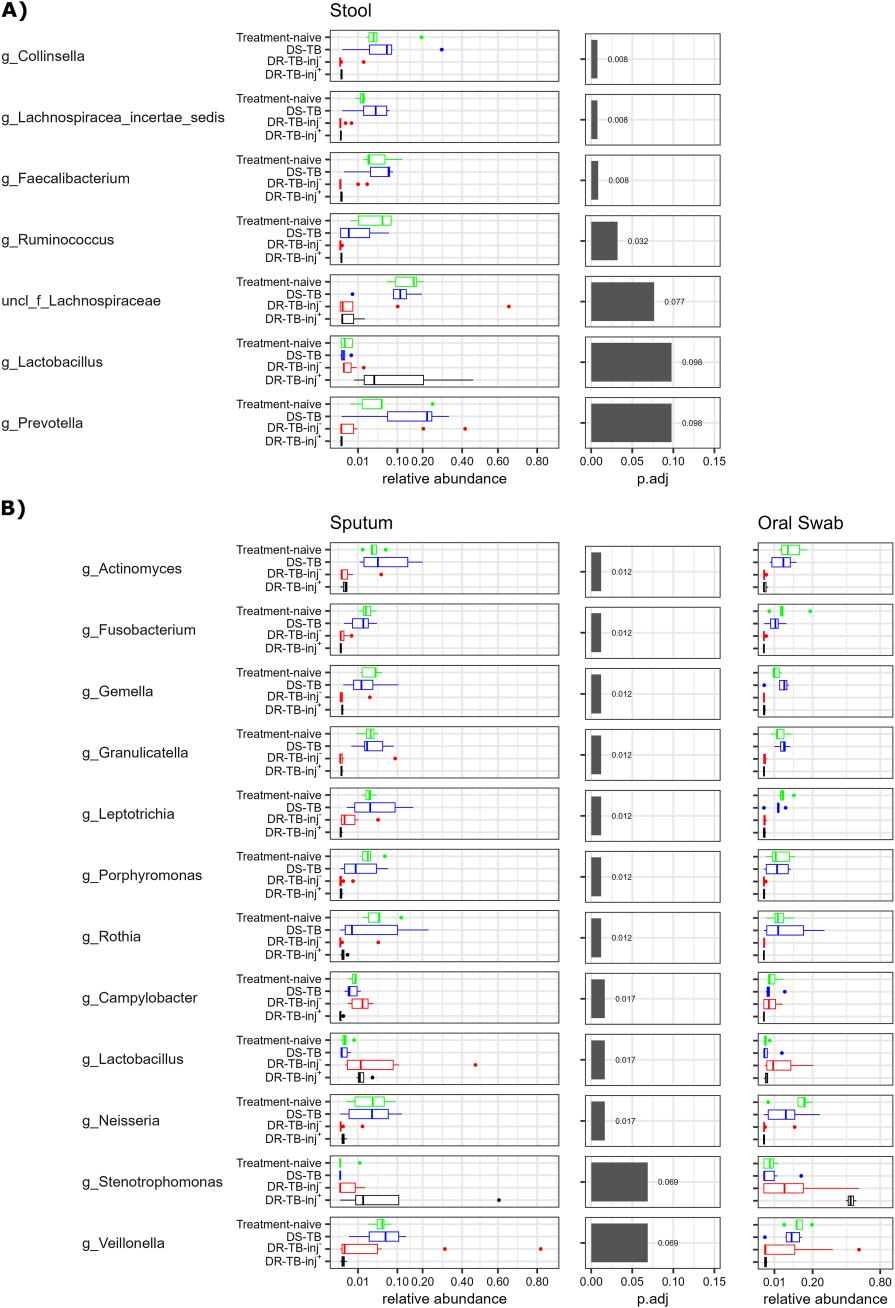
Differentially abundant taxa. Differential abundance of the 20 most abundant genera in **A)** stool and **B)** sputum at 27–42 days post-treatment start was estimated using the Kruskal-Wallis test, followed by Benjamini-Hochberg correction. Only taxa with corrected *P* < 0.1 are shown. Abundances in oral swab samples are shown as reference for the differential abundant taxa in sputum samples. Data from 5 participants with treatment-naïve TB, 6 participants with DS-TB, 10 participants with DR-TB-inj-, and 3–4 participants with DR-TB-inj+ are shown. DS-TB = drug-susceptible TB; DR-TB-inj– = drug-resistant TB treated without injectable agents; DR-TB-inj+ = drug-resistant TB treated with injectable agents (meropenem and/or amikacin).

Differentially abundant genera were identified among the 20 most abundant ones in stool and sputum samples at 27–42 days after treatment start (Figure 3 and Supplementary Figure S3) with *P* values of Wilcoxon signed rank tests summarised in Supplementary Table S1. Differences between non-treated participants and those receiving treatment for susceptible TB were minor, with observable but non-significant changes in the abundances of the genera *Ruminococcus* and *Prevotella*.

*Collinsella* (phylum Actinobacteria), members of the Firmicutes (Lachnospiraceae (Clostridiales), *Faecalibacterium* (Clostridium cluster IV), and *Ruminococcus*), and *Prevotella* (phylum Bacteroidetes) were almost lost from the stool microbiota in all the participants with DR-TB, either treated with all-oral or injectable drug regimens, when compared to treatment-naïve participants or those receiving treatment for DS-TB. Although non-significantly, the abundance of *Lactobacilli* (Firmicutes) was increased in specimens from participants in the DR-TB-inj+ group when compared to those from treatment-naïve ones, participants in the DS-TB group, or those in the DR-TB-inj– group (all *P* = 0.068).

**Figure 4.**
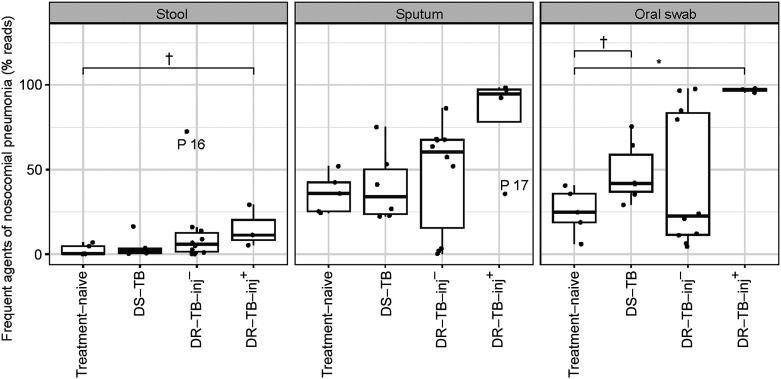
Cumulative abundances of taxa that are frequently associated with nosocomial pneumonia. The abundances of the genera *Pseudomonas, Stenotrophomonas, Streptococcus, Staphylococcus, Mycoplasma, Legionella, Klebsiella, Haemophilus* and of the *Enterobacteriaceae* family were cumulated and plotted for each treatment group. Statistical analyses were done using Wilcoxon tests. The outliers (P16, stool and P17, sputum) are indicated in the graph (they were not excluded from the statistical analysis). ^†^*P* < 0.1 * *P* < 0.05. DS-TB = drug-susceptible TB; DR-TB-inj– = drug-resistant TB treated without injectable agents; DR-TB-inj+ = drug-resistant TB treated with injectable agents (meropenem and/or amikacin).

In sputum, we found a significant reduction in the abundance of members of the phyla Actinobacteria (*Actinomyces*, *Rothia*), the Fusobacteria (*Fusobacterium*, *Leptotrichia*), the Firmicutes (*Gemella*, *Granulicatella*, *Veillonella*), and the Bacteroidetes (*Porphyromonas*) in participants of the DR-TB-inj– or DR-TB-inj+ groups, when compared to treatment-naïve ones or participants with DS-TB (Figure 3). Members of the phylum Proteobacteria showed either reduced abundances in the groups mentioned above (*Neisseria*), reduced abundance only in participants in the DR-TB-inj+ group (*Campylobacter*), or even increased abundance in sputa of participants treated for DR-TB (*Stenotrophomonas*, *P* = 0.14 for DR-TB-inj+ compared to participants with treatment-naïve TB). *Lactobacillus* (Firmicutes) represents the genus with the highest abundance increase in participants of the DR-TB group when compared to participants with treatment-naïve TB (*P* = 0.043 for DR-TB-inj–, *P* = 0.043 for DR-TB-inj+). Other genera that filled the niches were variable among participants and included commensal ones like *Veillonella*, but also many phylogenetically different pathobionts, including, for example, *Pseudomonas*, *Staphylococcus*, or *Mycoplasma* (Supplementary Figure S3). Some of the taxa with increased abundance fall into a group of bacteria that are frequently associated with nosocomial lung infection, including the genera *Pseudomonas*, *Stenotrophomonas*, *Streptococcus*, *Staphylococcus*, *Mycoplasma*, *Legionella*, *Klebsiella*, and *Haemophilus*, as well as the family Enterobacteriaceae.^14^ Although a significant difference in the nosocomial infection-associated bacteria can be seen only between the treatment-naïve and the DR-TB-inj+ groups in oral swab samples (*P* < 0.05), a trend towards increased abundance of these taxa along a gradient of increased treatment intensity can be seen in all sample-types. Notably, Patient 17, the only case that received delamanid and amikacin, had a lower relative abundance of nosocomial infection-associated taxa in the sputum than the other participants who received DR-TB-inj+ treatment (Figure 4). Differences between treatment groups in oral swab microbiota revealed the same trends observed in sputum samples. However, the increase of *Stenotrophomonas* in the DR-TB-inj+ group was more pronounced in oral swabs compared to the one seen in sputum samples, while *Lactobacillus* was slightly more abundant in sputa than in oral swabs (Figure 3).

## DISCUSSION

We investigated changes in the stool, sputum, and oral swab microbiota of persons with pulmonary TB before treatment and 4–6 weeks following treatment initiation with different anti-TB treatment regimens. Along a gradient of intensified anti-TB treatment, alpha diversity of the microbiota decreased in all compartments. Further, we observed relative increases of *Lactobacillus* and other genera, which are heterogeneous between participants and phylogenetically diverse. Especially in the respiratory tract, these include genera, including *Stenotrophomonas*, *Pseudomonas*, *Staphylococcus*, and *Mycoplasma*, that contain well-known pathobionts frequently associated with nosocomial pneumonia. Microbiota compositions in sputum and oral cavity are almost similar in the treatment-naïve, DS-TB, and DR-TB-inj– groups, while slight differences exist in the DR-TB-inj+ group.

Treatment for DS-TB had a relatively small impact on the alpha diversity of stool samples in our study. This is in line with previous studies comparing stool microbiota compositions from treatment-naïve cases with either latent or active TB and those receiving standard treatment with isoniazid, rifampicin, pyrazinamide, and ethambutol.^15–19^ Our results also show a slight to moderate decrease in alpha diversity in sputum samples of participants treated with a standard anti-TB regimen. Previous reports show controversial data for the respiratory tract microbiota, ranging from decreased^20–22^ to increased^23,24^ alpha diversities. Differences are possibly explained by the selection criteria (one study reported increased alpha diversity after 2 months of anti-TB treatment and bronchoalveolar lavage smear and culture-negative conversion^24^), type of sampling (i.e. sputum vs. bronchoalveolar lavage), sequencing technology, or differences in the study populations. The limited impact of the standard anti-TB treatment on the microbiota composition is in line with the narrow target range of isoniazid, ethambutol, and pyrazinamide. However, the low impact of rifampicin is surprising as its target enzyme, the DNA-dependent RNA polymerase, represents a successful drug target in other infections by both Gram-negative and Gram-positive bacterial pathogens.^15,25,26^

In contrast, treatment with second-line anti-TB treatment leads to decreased alpha diversity and community shifts in the stool microbiota in our study, especially when injectable aminoglycosides and/or carbapenems are included (i.e. in the DR-TB-inj+ group). This is consistent with the strong and long-lasting effects described for DR-TB previously.^12^ A smaller decrease in alpha diversity has been described in a study where controls were not treatment-naïve but received standard anti-TB treatment for DS-TB.^13^ To our knowledge, this is the first study comparing stool microbiota diversities in persons with TB treated with different types of second-line anti-TB drugs. Our results also show significantly reduced alpha diversity in the respiratory tract of persons treated for DR-TB, again most drastically when aminoglycosides and/or carbapenems are included (i.e. in the DR-TB-inj+ group). To our knowledge, this is also the first study investigating the impact of second-line anti-TB drug regimens on the respiratory microbiota.

We observed decreased abundances of a genus of Lachnospiraceae, *Collinsella*, *Faecalibacterium*, and *Ruminococcus* in the stool of participants treated for DR-TB. The decreased *Collinsella* and *Faecalibacterium* are in line with a previous report,^12^ and the reduced member of Lachnospiraceae is consistent with a reported reduction of the genus *Blautia*.^12^ However, to our knowledge, reducing the genus *Ruminococcus* is a new finding. By trend, the *Lactobacillus* was increased in the DR-TB-inj+ group in the stool. We did not find increased Proteobacteria in stool samples of participants treated for DR-TB, which is in contrast to a previous report.^13^ We observed the strongest effects on the respiratory microbiota after DR-TB-inj+ treatment. All drug regimens in this group included meropenem, which confers a broad antibacterial spectrum.^27^ We identified two genera, *Lactobacillus* and *Stenotrophomonas*, with increased abundance during treatment with DR-TB regimens. Notably, the pathobiont *Stenotrophomonas maltophilia*, which has been described to carry multiple drug resistances, including resistance against meropenem and amikacin, has been identified upon antibiotic treatment of other infectious diseases such as cystic fibrosis.^28,29^ Genera that filled the niches were variable among participants and included many phylogenetically different pathobionts, including *Pseudomonas*, *Staphylococcus*, or *Mycoplasma*, which are frequently associated with nosocomial pneumonia. In line with this observation, a case of *Pseudomonas* and *Klebsiella* growth in the sputum culture of a patient treated for XDR-TB has been reported recently^30^ (personal communication C Lange). It is conceivable that in such cases, pneumonia-associated taxa contribute to life-threatening pathogenesis, including sepsis.

The sputum and oral cavity microbiota show comparable patterns in the treatment-naïve, DS-TB, and DR-TB-inj– groups. This is expected since the oral cavity is tightly linked with the lower respiratory tract and harbours a dense microbial population, while the lower respiratory tract contains relatively few microbes.^31^ It is also well established that microbes in the oral cavity are inhaled and thus shape the composition of the lower respiratory tract microbiota, at least in healthy subjects, where immigration and elimination factors determine their composition.^32^ The higher abundance of *Stenotrophomonas* in the DR-TB-inj+ group in oral swabs compared to sputum samples is remarkable, even considering that one oral swab sample is missing in the analysis due to low 16S rRNA sequencing coverage. Therefore, other taxa must exist, which have higher relative abundance in the sputum than the oral swab microbiota, indicating that local niches have formed in the DR-TB-inj+ group, enabling microbial growth in the lower respiratory tract. However, this hypothesis is speculative, given the limited sample size in the DR-TB-inj+ group investigated in this study.

A limitation of this study is that only data from one sampling time point existed for most participants. Therefore, changes in the microbiota before and during treatment could not be compared in individual participants. Follow-up samples post 6 weeks of treatment were not available, precluding the analysis of long-term microbiota alterations. Further limitations are the limited patient numbers, the diversity in the treatment regimens among the patients with DR-TB, and the use of sputum instead of bronchoalveolar lavage specimens. As 16S sequencing was used, the data were limited to the bacterial microbiota. The strengths of this study are the inclusion of persons with treatment-naïve active tuberculosis alongside three groups that represent increasing intensity of anti-tuberculosis treatment. Further, three sampling sites were included, representing intestinal, lower- and upper-respiratory tracts.

In conclusion, standard anti-TB treatment of persons with DS-TB may be associated with limited alterations of stool, respiratory tract, and oral cavity microbiota within the first 4–6 weeks of therapy. However, treatment against DR-TB, especially when aminoglycosides and/or carbapenems are included, leads to low diversity in stool microbiota at 6 weeks of anti-TB treatment. In persons treated for *M. tuberculosis* with increased levels of drug resistance, the relative abundance of pathobionts increased in the respiratory tract, bearing the risk of tuberculosis disease exacerbation and sepsis.

## Supplementary Material


