# Supplementary material for: Microbiota alterations in patients treated for susceptible or drug-resistant TB
Source: IJTLD Open. 2024 Aug 1;1(8):355–61. doi: 10.5588/ijtldopen.24.0325 (PMC11308407; doi:10.5588/ijtldopen.24.0325)
Supplement: Supplementary file 1 [file ijtldopen0325_supplementarydata1.pdf]

http://dx.doi.org/10.5588/ijtldopen.24.0325

Microbiota alterations in patients treated for susceptible or drug-resistant TB

SUPPLEMENTARY DATA

Sample collection

Participants that were recruited before treatment start were asked to provide oral swab, sputum, and stool samples before initiation of anti-tuberculosis therapy. All participants were asked to provide samples at 27-42 days post initiation of DS-TB, DR-TB-inj$^-$, or DR-TB-inj$^+$ treatment, respectively. Stool and sputum were collected in DNAse/RNAse-free tubes. Flocked swabs (Zymo-Research, Irvin, CA, USA) were used to take samples from the oral cavity. Swabs were placed into RNA/DNA Shield (Zymo-Research, Irvin, CA, USA). Sputum samples were incubated with sputolysin for homogenization and centrifuged to sediment bacteria. The pellets were suspended in TRI-Reagent (Zymo-Research, Irvin, CA, USA) for pathogen inactivation and DNA-extraction. 50-100 mg of stool samples were suspended in 1 ml TRI-Reagent. 300 µl of RNA/DNA Shield containing oral swab specimens were mixed with 1 ml TRI-Reagent. At all stages, samples were stored at -80°C.

16S rRNA gene sequencing

Pre-PCR samples were handled at dedicated workplaces and with pipettes that were regularly decontaminated from nucleic acids. Molecular Biology grade disposables were used. DNA was isolated using a back-extraction protocol (Thermo Fisher Scientific, Waltham, Massachusetts). Briefly, samples were homogenized by 5x50 s beat beating. Chloroform was added, the mixture was centrifuged, and the organic phase containing DNA was isolated. Then, back extraction buffer (4 M guanidine thiocyanate, 50 mM sodium citrate, 1 M Tris) was added, the mixture was centrifuged, and the aqueous phase containing DNA was isolated. Acrylamide was added as DNA carrier. DNA was pelleted with Isopropanol, washed with 75% ethanol, and resuspended in 8 mM NaOH before the pH was adjusted with HEPES-buffer. DNA was further purified using magnetic beads (MagnaMedics, Aachen, Germany). The 16S rRNA V3-V4 region was amplified by 25 PCR-cycles using standard 341-fw (CCTACGGGNGGCWGCAG)[1] and 806-rv (GGACTACHVGGGTWTCTAAT)[2] primers, elongated by Illumina overhang adapter sequences (Eurogentec, Seraing, Belgium). Amplification was performed with 10 ng (stool) or 40 ng (sputum, oral swab specimens) of DNA-template and Phusion High Fidelity polymerase (Thermo Fisher Scientific, Waltham, Massachusetts). DNA-cleanup was performed using magnetic beads, index-PCR was performed with 8 PCR-cycles (stool) or 10 cycles (sputum, oral swab) followed by Illumina's 16S rRNA sequencing protocol using an Illumina MiSeq v3 2x300bp kit (Illumina, San Diego, CA, USA).

Raw data were analyzed with USEARCH 64-bit (v10). Briefly, sequences were truncated at a q score of 30, and paired sequences were merged allowing a minimum overlap length of 40 bp. To build an operational taxonomic unit (OTU) stability file, sequences were filtered to obtain unique sequences

with a minimum length of 330 bp and a maximum of 1 expected error. OTU-clustering was performed using the function “-cluster_otus”, which includes chimera removal. An OTU-table was created based on 97% sequence identity and taxonomic assignment was performed with 80% cutoff.

Statistics

Participants with incomplete sample-sets before treatment initiation were excluded for the pre-treatment timepoint. Analysis was performed using phyloseq (v. 1.40.0) and vegan (v. 2.6-2) of R (v. 4.2.2, R Core Team, 2022) in an R Studio (v. 2022.07.1) environment. Samples with <9,240 reads were excluded. Relative abundance data were used in further analyses. The Shannon index was employed as an indicator of alpha diversity. Overall shifts in microbiota community compositions were analyzed by employing the Bray-Curtis dissimilarity index and visualizing using PCoA plots. Taxon-abundances were visualized in heatmaps. Differential abundances were identified using Kruskal-Wallis test for the main group-effect and Wilcoxon tests for the pairwise comparisons of groups. Benjamini-Hochberg correction was used to account for multiple comparisons. Permutational ANOVA was performed using the Adonis function of vegan.

References

1. Klindworth A, et al., *Evaluation of general 16S ribosomal RNA gene PCR primers for classical and next-generation sequencing-based diversity studies.* Nucleic Acids Research, 2012. **41**(1): p. e1-e1.
2. Caporaso JG, et al., *Global patterns of 16S rRNA diversity at a depth of millions of sequences per sample.* Proc Natl Acad Sci U S A, 2011. **108 Suppl 1**(Suppl 1): p. 4516-22.

SUPPLEMENTARY TABLES

Suppl. Table 1: Summary of p-values as calculated for pairwise Wilcoxon signed rank tests with Benjamini-Hochberg correction of differentially abundant taxa in stool, sputum, and oral swab samples.

a) stool

Genus	treat.naive : DS-TB	treat.naive : DR-TB-inj⁻	treat.naive : DR-TB-inj⁺	DS-TB : DR-TB-inj⁻	DS-TB : DR-TB-inj⁺	DR-TB-inj⁻ : DR-TB-inj⁺
**uncl_f_Lachnospiraceae**	0,56	0,068	0,068	0,068	0,083	0,73
**g_Lactobacillus**	0,68	0,73	0,068	0,18	0,068	0,068
**g_Prevotella**	0,64	0,13	0,068	0,068	0,083	0,93
**g_Collinsella**	0,81	0,018	0,068	0,018	0,068	0,26
**g_Faecalibacterium**	0,81	0,019	0,068	0,019	0,068	0,64
**g_Ruminococcus**	0,18	0,018	0,068	0,15	0,64	0,33

**g_Lachnospiracea_incertae_sedis**	0,45	0,018	0,068	0,018	0,068	0,73

b) sputum

Genus	treat.naive : DS-TB	treat.naive : DR-TB-inj$^-$	treat.naive : DR-TB-inj$^+$	DS-TB : DR-TB-inj$^-$	DS-TB : DR-TB-inj$^+$	DR-TB-inj$^-$: DR-TB-inj$^+$
**g_Veillonella**	0,86	0,19	0,043	0,21	0,043	0,58
**g_Stenotrophomonas**	0,46	0,71	0,14	0,17	0,05	0,14
**g_Lactobacillus**	0,75	0,043	0,043	0,046	0,043	0,96
**g_Rothia**	0,53	0,043	0,043	0,043	0,2	0,18
**g_Actinomyces**	0,86	0,043	0,043	0,043	0,043	0,96
**g_Leptotrichia**	1	0,043	0,043	0,043	0,043	0,17
**g_Neisseria**	0,96	0,043	0,059	0,05	0,24	0,19
**g_Gemella**	0,44	0,043	0,043	0,043	0,043	0,46
**g_Granulicatella**	0,96	0,043	0,043	0,043	0,043	0,37
**g_Porphyromonas**	0,44	0,043	0,043	0,052	0,17	0,96
**g_Fusobacterium**	0,64	0,043	0,043	0,043	0,043	0,7
**g_Campylobacter**	0,75	0,47	0,043	0,16	0,043	0,043

c) oral swab

Genus	treat.naive : DS-TB	treat.naive : DR-TB-inj$^-$	treat.naive : DR-TB-inj$^+$	DS-TB : DR-TB-inj$^-$	DS-TB : DR-TB-inj$^+$	DR-TB-inj$^-$: DR-TB-inj$^+$
**g_Veillonella**	0,46	0,28	0,08	0,39	0,18	0,69
**g_Stenotrophomonas**	0,98	0,41	0,08	0,46	0,08	0,26
**g_Rothia**	1	0,018	0,08	0,073	0,17	0,65
**g_Neisseria**	0,37	0,02	0,08	0,098	0,26	0,85
**g_Lactobacillus**	0,75	0,08	0,95	0,08	1	0,28
**g_Fusobacterium**	0,28	0,018	0,08	0,018	0,08	0,48
**g_Actinomyces**	0,46	0,018	0,08	0,018	0,08	1
**g_Porphyromonas**	0,85	0,018	0,08	0,1	0,35	1
**g_Leptotrichia**	0,23	0,018	0,08	0,073	0,17	0,85
**g_Granulicatella**	0,66	0,018	0,08	0,018	0,08	0,65
**g_Gemella**	0,28	0,018	0,08	0,018	0,11	0,85
**g_Campylobacter**	0,55	0,55	0,08	0,74	0,08	0,37

SUPPLEMENTARY FIGURES

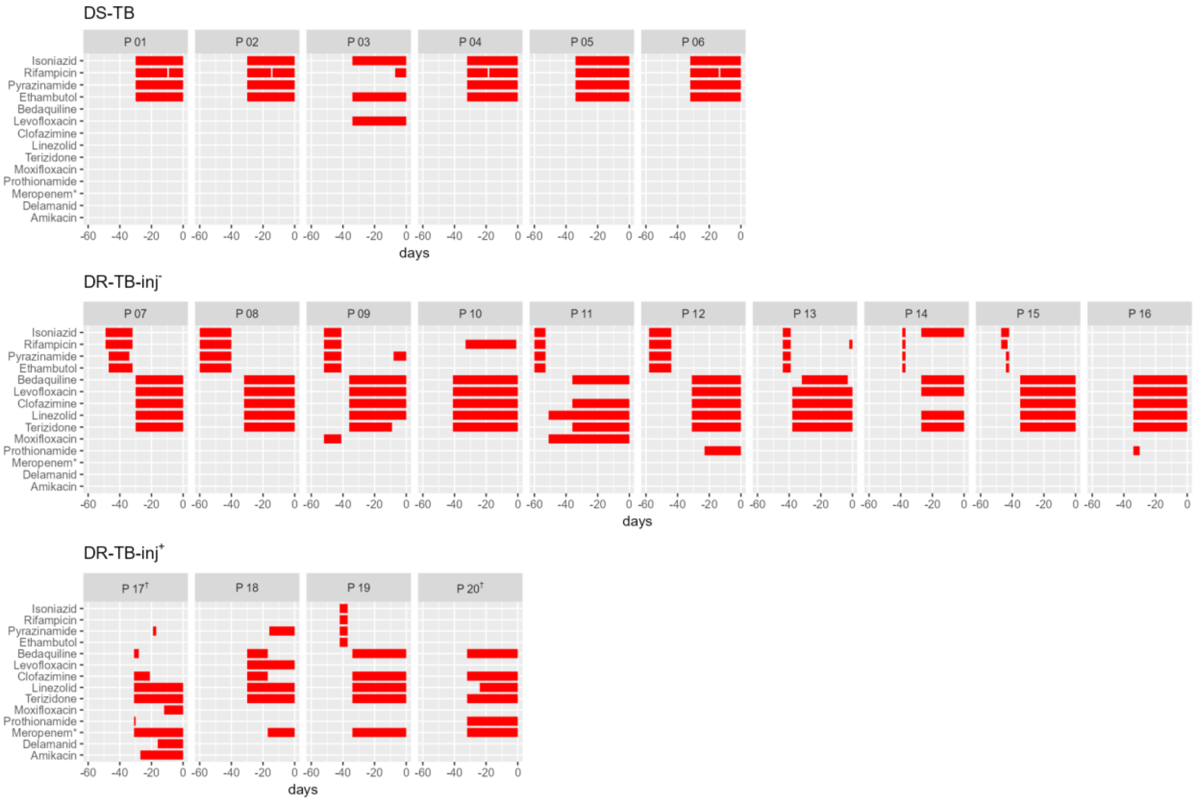

Suppl. Figure 1: Treatment history of individual patients. 6 patients received treatment for susceptible TB (DS-TB), 10 patients received all-oral treatment for MDR/RR-TB (DR-TB–inj^{-}) and 4 patients received injectable drug regimens for MDR/RR-TB (DR-TB-inj^{+}). As indicated, patients provided samples at 27-42 days post treatment start or post treatment change, respectively. 5 patients (P2, P6, P10, P17, P18) provided samples before treatment start, which were used as treatment-naïve controls. Stool, sputum, and oral swab samples were collected and analyzed for the bacterial microbiota. *Patients receiving meropenem also received amoxicillin and clavulanic acid. †Stool microbiota not determined from P17, and oral swab microbiota not determined from P20.

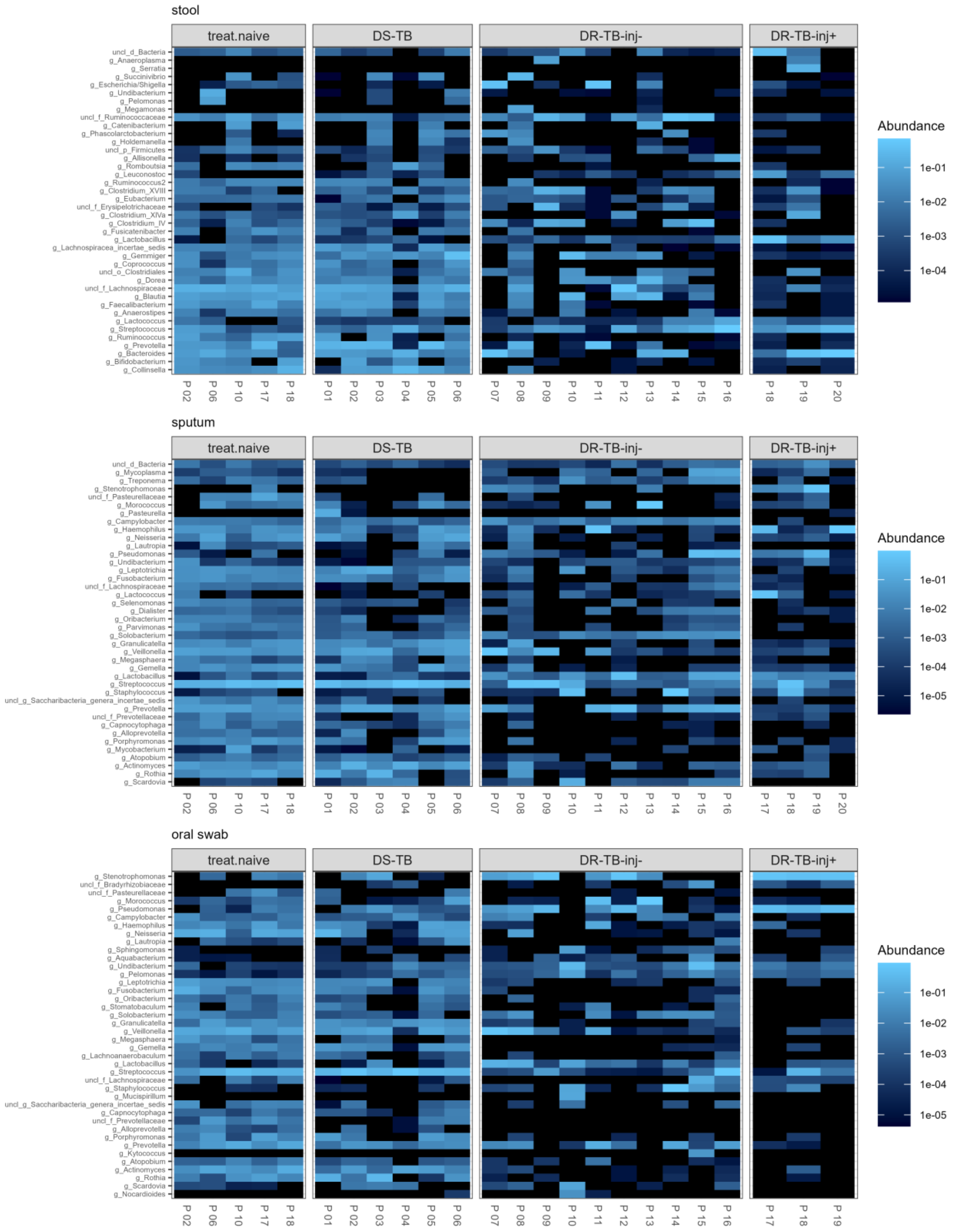

Suppl. Figure 2: Abundance of most abundant genera. The 40 most abundant genera in stool, sputum, and oral swab of the individual patients shown as heat map.

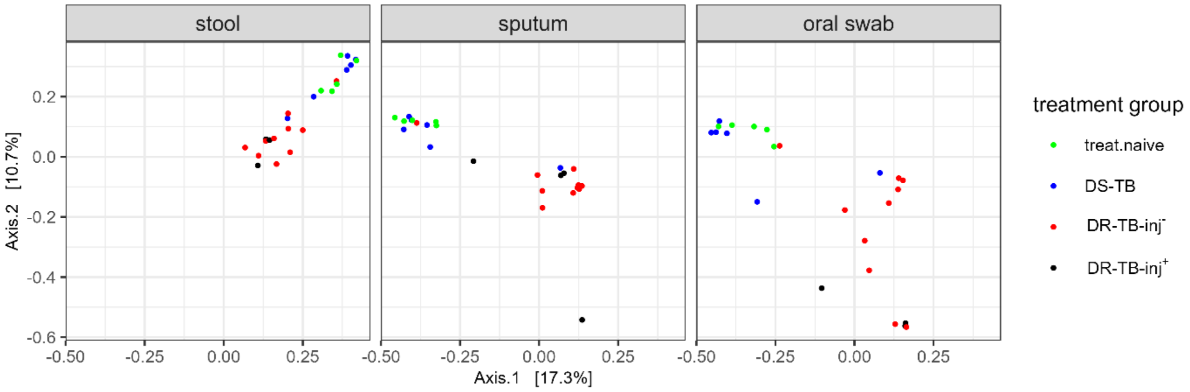

Suppl. Figure 3: Beta diversity. Principal Coordinate Analysis of Bray Curtis dissimilarity indices in stool, sputum, and oral swab specimens. The plots illustrate similarities and dissimilarities of the microbiota compositions of treatment-naïve participants with TB and those treated for DS-TB, DR-TB–inj$^-$, or DR-TB-inj$^+$. Statistical comparisons by permutational ANOVA revealed significant differences between microbiota compositions of the different treatment groups in stool, sputum, and oral swab (all $p<0.001$). n=5 (treatment-naïve), n=6 (DS-TB), n=10 (DR-TB–inj$^-$), n=3-4 (DR-TB-inj$^+$).
